# Description of an Integrated and Dynamic System to Efficiently Deal With a Raging COVID-19 Pandemic Peak

**DOI:** 10.3389/fmed.2022.819134

**Published:** 2022-03-18

**Authors:** Vanni Agnoletti, Emiliano Gamberini, Alessandro Circelli, Costanza Martino, Domenico Pietro Santonastaso, Giuliano Bolondi, Giorgia Bastoni, Martina Spiga, Paola Ceccarelli, Luca Montaguti, Fausto Catena, Venerino Poletti, Carlo Lusenti, Claudio Lazzari, Mattia Altini, Emanuele Russo

**Affiliations:** ^1^Department of Emergency Surgery and Trauma, Anesthesia and Intensive Care Unit, M Bufalini Hospital, Cesena, Italy; ^2^Nursing Department, M Bufalini Hospital, Cesena, Italy; ^3^Department of Internal Medicine, M Bufalini Hospital, Cesena, Italy; ^4^Department of Emergency Surgery and Trauma, M Bufalini Hospital, Cesena, Italy; ^5^Department of Respiratory Diseases, AUSL Romagna-Morgagni Hospital, Forlì, Italy; ^6^Hospital Direction M Bufalini Hospital, Cesena, Italy; ^7^Hospital Direction Azienda Unità Sanitaria Locale della Romagna, Ravenna, Italy

**Keywords:** COVID-19, intensive care unit, high dependency unit, step-down unit, hospital admission criteria, bed management, bed occupancy rate, patients throughput

## Abstract

**Background:**

This study aimed to describe an innovative and functional method to deal with the increased COVID-19 pandemic-related intensive care unit bed requirements.

**Methods:**

We described the emergency creation of an integrated system of internistic ward, step-down unit, and intensive care unit, physically located in reciprocal vicinity on the same floor. The run was carried out under the control of single intensive care staff, through sharing clinical protocols and informatics systems, and following single director supervision. The intention was to create a dynamic and flexible system, allowing for rapid and fluid patient admission/discharge, depending on the requirements due to the third Italian peak of the COVID-19 pandemic in March 2021.

**Results:**

This study involved 142 COVID-19 patients and 66 non-COVID-19 patients who were admitted; no critical patient was left unadmitted and no COVID-19 severe patients referring to our center had to be redirected to other hospitals due to bed saturation. This system allowed shorter hospital length-of-stay in general wards (5.9 ± 4 days) than in other internistic COVID-19 wards and overall mortality in line with those reported in literature despite the peak raging.

**Conclusion:**

This case report showed the feasibility and the efficiency of this dynamic model of hospital rearrangement to deal with COVID-19 pandemic peaks.

## Introduction

Since its onset, the COVID-19 pandemic has pressured healthcare systems worldwide. We previously described a functional and dynamic strategy that allowed our intensive care unit (ICU) to deal with the first two peaks of the pandemic, in March and October 2020, that in our community reached 8.2 cases per 1,000 inhabitants (1).

The setting is a public hospital of 450 beds, which is a reference point for a population of about 210,000 people. Before COVID-19, the ICU consisted of 18 beds, whereas during the pandemic, 6 non-COVID ICU beds and 9 step-down unit (SDU) beds were opened.

This case study aimed to show how this dynamic system has been furtherly implemented to deal with the third and strongest pandemic peak, in March 2021, reaching a local incidence of 13.3 positive cases per 1,000 inhabitants (62% increase).

## Methods

As the third peak was arising, all the hospital non-COVID wards were promptly resized and relocated. Strategically, the first units left empty were those close to the ICU-SDU (4^th^ floor) and to the COVID-internal medicine (6^th^ floor).

**Figure 1:**
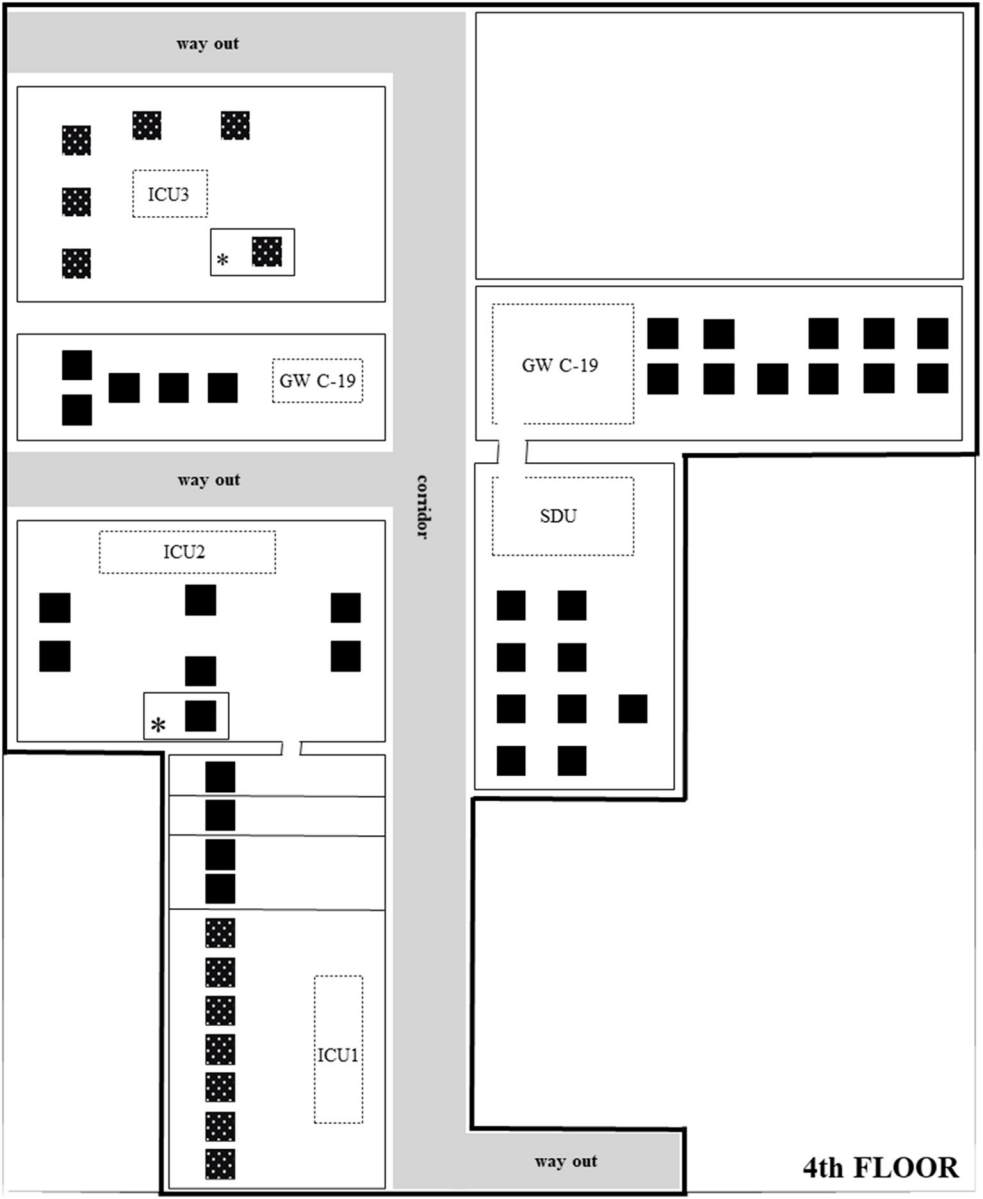
Critical area on the 4th floor. Planimetry and bed location of the 4^th^ floor, under the direct control of the intensive care department. Full-black beds,COVID-19 patients; Spotted-black beds, non-COVID-19 patients; Asterick, supra-numerary beds, usually not in use, open only for emergency; Dotted rectangles with unit name, medical positions and monitor areas physically separated from patient's open-space.

With respect to the previous strategy, a further internistic COVID unit, General Internal Medicine Ward COVID-19 (GW_C19_), of 16 beds was carved out on the 4^th^ floor, next to the SDU (Figure 1). It was staffed by an intensivist physician, with an 8:1 patient-per-nurse ratio (PPNR), wherein nurses were not ICU-trained. Two monitored beds (oxygen saturation, non-invasive or invasive blood pressure, and ECG) for patients needing high flow nasal cannulae (HFNC) were available. Admission criteria to this general ward were the following: patients positive to severe acute respiratory syndrome coronavirus 2 (SARS-CoV-2), with radiologic evidence of pulmonary interstitiopathy, dyspnea, oxygen saturation (SpO_2_) ≥ 92% on room air, and low oxygen supplementation (less 10 L/min). Clinical factors, such as fever, hypertension, and diabetes mellitus, were considered risk factors for possibly severe evolution (2).

The 4th floor, under the control of the intensive care department, thus counted on the following: 16 GW_C19_ beds, 1 intensivist, 8:1 PPNR; 9 beds SDU, 1 intensivist, and 3:1 PPNR (at least 1 ICU-trained nurse); 11 ICU1 beds, 2 intensivists (1 during the night shift), and 2:1 PPNR; 7 ICU2 beds, 2 intensivists (1 during the night shift), and 2:1 PPNR; 6 ICU3 beds, 1 intensivist, and 2:1 PPNR (Figure 1). Admission criteria to SDU were previously described (1).

This was made possible by a drastic 50% reduction of the planned operating room activity, actuated for safety reasons, that allowed to recover a sufficient number of anesthetists to be employed at the 4^th^ floor. In Italy, anesthesia and intensive care still constitute a single residency program, thus specialists are certified and trained to manage complex patients with one or more ongoing organ failure.

In March 2021, the Italian Ministry of Health published new guidelines on the management of isolation of severe COVID-19 patients: (3) 21 days after the first SARS-CoV-2 positivity, if asymptomatic or with a negative molecular test, they were considerable non-infective and transferable to non-COVID units. This increased the fluidity in the management of bed occupancy.

## Results

In March 2021 (31 days), a total of 142 COVID-19 patients and 66 non-COVID-19 patients were admitted to the 4^th^ floor. Table 1 describes the characteristics of these patients. The 4th-floor setting allowed to admit every patient to a level of intensiveness appropriate for their clinical status.

**Table 1:** Clinical description of the patients admitted in the different units during the pandemic peak.

	**GW_**C19**_**	**SDU**	**ICU_**C19**_**	**ICU_**NO-C19**_**
Number of patients admitted	76	41	25	66
Men/women	46/30	28/13	19/6	36/30
Mean (SD) age, in years	63.7 (9.2)	61.4 (12.5)	58.3 (12.4)	61.8 (13.4)
Mean (SD) length-of-stay (LOS), in days	5.9 (4.0)	7.0 (4.6)	8.6 (6.7)	4.0 (5.3)

*GW_C19_, General (Internal Medicine) Ward COVID-19; SDU, Step Down Unit; ICU_C19_, Intensive Care Unit COVID-19; ICU_no−C19_, Intensive Care Unit Non-COVID-19*.

Mean length-of-stay (LOS) (*SD*) in days was 5.9 (4) for GW_C19_, 7 (4.6) for SDU, 8.6 (6.7) for ICU COVID-19 (ICU_C19_), and 4 (5.3) for ICU non-COVID-19 (ICU_no−C19)_. The mortality rate was 2.6% for GW_C19_, 20% for SDU, 36% for ICU_C19_, and 9.2% for ICU_no−C19_, in line with available literature (4, 5).

**Figure 2:**
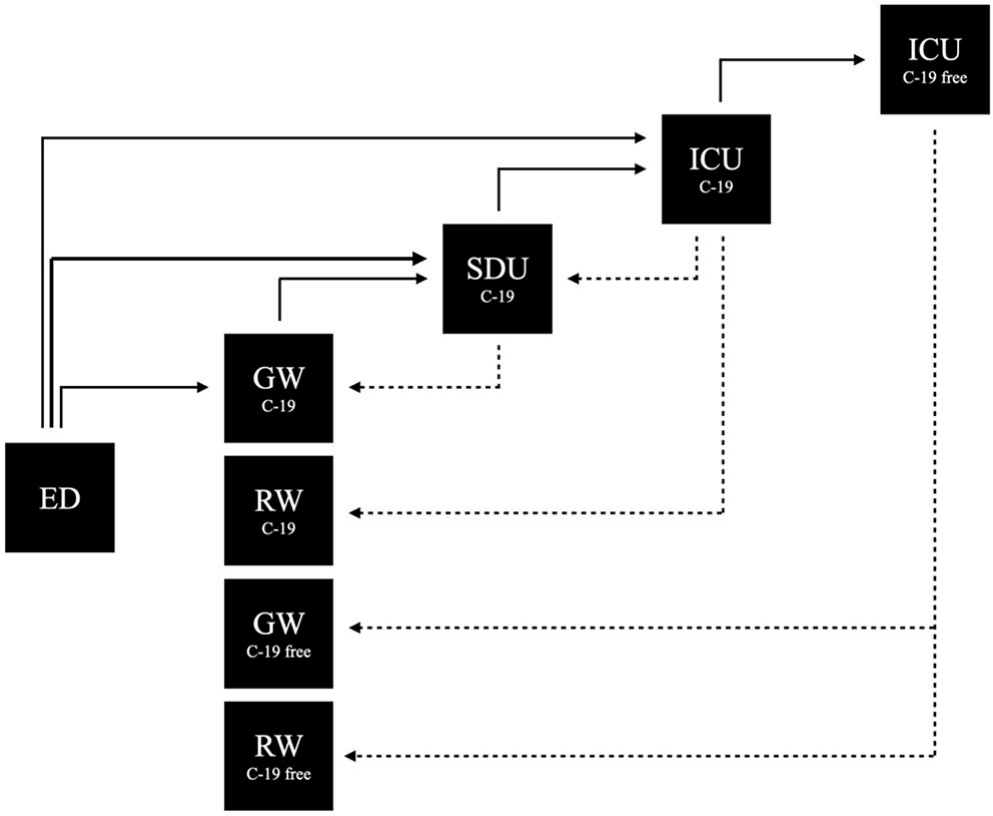
Patient COVID-19 flow. Patients flow between the different units during the pandemic peak. For the number of patients refere to Table 2. ED, Emergency department; GW C-19, General ward COVID-19; RW C-19, Rehabilitation ward COVID-19; SDU C-19, Step down unit COVID-19; ICU C-19, Intensive care unit COVID-19; ICU C-19 free, Intensive care unit no-COVID-19; GW C-19 free, General ward no-COVID-19; RW C-19 free, Rehabilitation ward no-COVID-19.

Table 2, Figure 2 summarize the overall flow of patients between different units.

**Table 2:** Summary of the provenience and the discharge destination of all the patients treated at the 4^th^ floor integrated system led by the intensive care department.

**4^**TH**^ floor unit**	**GW_**C19, N (%)**_**	**SDU, _**N (%)**_**	**ICU_**C19, N (%)**_**	**ICU_**NO-C19, N (%)**_**
**Patients coming from (admission):**				
**COVID-19 wards**				
ED	41 (53.9)	8 (19.5)	1 (4)	19 (28.8)
GW_C19_	-	26 (63.4)	8 (32)	-
GeW_C19_	24 (31.5)	-	-	-
SDU	11 (14.4)	3 (7.3)	11 (44)	1 (1.5)
ICU_C19_	-	4 (9.7)	-	-
oICU_C19_	-	-	5 (20)	-
**Non-COVID-19 wards**				
GW_no−C19_	-	-	-	40 (60.6)
ICU_no−C19_	-	-	-	6 (9.1)
**Total number (%)**	76 (100)	41 (100)	25 (100)	66 (100)
**Patients going to (discharge):**				
**COVID-19 wards**				
GW_C19_	-	20 (42.5)	-	-
oGW_C19_	9 (14.2)	6 (12.7)	1 (7.4)	-
SDU	10(15.9)	-	7 (50)	-
ICU_C19_	-	11 (23.4)	-	-
oICU_C19_	-	10 (21.2)	5(35.7)	-
RW_C19_	-	-	1 (7)	-
**Non-COVID-19 wards**				
GW_no−C19_	-	-	-	44 (63.8)
ICU_no−C19_	-	-	-	9 (13)
RW_no−C19_	-	-	-	16 (23.2)
Home	44 (69.8)	-	-	-
**Total number (%)**	63 (100)	47 (100)	14 (100)	69 (100)

*The upper part of the table shows from where the patients were admitted to the different units. The lower part of the table shows to which units the patients were discharged. oICU_C19_ refer to nearby hospital ICUs collapsed during the pandemic peak, which referred 5 of their supernumerary patients to our ICU_C19_. Other patients were carried from the ambulances directly from nearby provinces to our emergency department. As soon as they were stabilized and a bed was available in their local ICU or hospital, they were discharged outside our hospital. A geriatric ward (GeW_C19_) was also available for elderly patients in our hospital, although not described in the paper. When a patient fulfilled the admission criteria, he was admitted to the GW_C19_ despite the advanced age, upon bed availability. ED, Emergency Department; GW_C19_, General (Internal Medicine) Ward Covid-19; oGW_C19_, Other General (Internal Medicine) Ward Covid-19 not led by intensivists; GeW_C19_, Geriatric Ward Covid-19, GW_no−C19_; SDU, Step Down Unit; ICU_C19_, Intensive Care Unit Covid-19; oICU_C19_, Other Intensive Care Unit Covid-19 in a different hospital; RW_C19_, Rehabilitation Ward Covid-19; GW_no−C19_, General Ward No Covid-19; ICU_no−C19_, Intensive Care Unit No Covid-19; RW_no−C19_, Rehabilitation Ward No Covid-19*.

Mean (± *SD*) LOS in GW_C19_ was shorter than in other internistic COVID-19 wards: 5.9 (4) days vs. 11.8 (5) days (*p* < 0.0001; unpaired *t*-test). This is probably due to the higher rapidity by which patients were transferred to a higher level of care through early detection of clinical deterioration and simple transfer systems or to the mindset of intensivists used to working with short timescales. All patients with severe COVID-19 who were referred to our hospital have been hospitalized, none needed to be referred to other hospitals, thanks to a system that avoided hospital bed saturation.

## Discussion

The decision to staff the GW_C19_ with anesthesiologists/intensivists was proposed by the hospital direction due to staff contingency. The director of anesthesia and ICU and the collaborators agreed with this setting, to ease and improve the management of patient discharge from ICU and SDU, trying to avoid bed saturation. Being part of the same team, sharing the same protocols, informatics system (Margherita 3), and coordinators allowed considerable time-saving. This was an efficient solution to maintain a safe and balanced hospital environment.

Differently from the previous report from March and April 2020, SDU worked more as a high dependency unit (HDU), at a semi-intensive care level, more complex than a common step-down unit (6). Furthermore, 8 patients were transferred from ICU_C19_ to SDU without requiring invasive ventilation; of the 41 patients admitted in SDU, only 11 needed escalation to ICU_C19_ for higher monitoring or orotracheal intubation; 34 patients were admitted directly from the emergency department (ED) to SDU. These data seem to testify to the high intensity of care reached in SDU at this third wave.

A limitation of this report is that, by its nature of case study, it is not matched with a comparative system. Moreover, at first superficial sight, the employment of intensivists in GW_C19_ and SDU might seem a waste of resources. In our experience, this has allowed many physicians to cyclically work at a lower intensity, periodically decompressing from the stress and pressure of a year in an ICU_C19_, interacting with conscious patients experiencing better outcomes, thus reducing burnout problems (7).

A further limitation of this system is that it worked in our specific context, whereas, it might not be applicable for hospitals acting as referral centers for a much wider general population and it might also not be applicable to regions where the incidence rate is much higher, determining a dramatic pandemic wave.

The model of differential intensity for hospital care management (high-intensity for ICU, medium-intensity for SDU, and low-intensity for GW), handled by a single intensive care unit, determined a sort of independence of the 4^th^ floor from the hospital. The 4^th^ floor was able to admit COVID-19 patients from other units/floors as needed from their clinical status evaluated by a consultant, but internally there was no need for hospital bed-manager coordination, counseling requests, bureaucracy, and time-wasting procedures.

## Conclusion

The use of a COVID-19 “critical floor” from the general ward to ICU is an example of system adaptability. The effort made by intensivists was useful for patients in terms of quality of care and doctors in terms of occupational stress and mental health.

## Data Availability Statement

The original contributions presented in the study are included in the article/supplementary material, further inquiries can be directed to the corresponding author.

## Author Contributions

VA conceived the study and wrote the article. EG, AC, CM, DS, and GBo worked in COVID-19 ICU and wards and helped to write the article. GBa, MS, and PC coordinated the nursing staff in the management of the ward. LM, FC, CLu, MA, CLa, and VP collaborated to review the manuscript. ER helped to write the manuscript and provided supervision. All authors contributed to the article and approved the submitted version.

## Conflict of Interest

The authors declare that the research was conducted in the absence of any commercial or financial relationships that could be construed as a potential conflict of interest.

## Publisher's Note

All claims expressed in this article are solely those of the authors and do not necessarily represent those of their affiliated organizations, or those of the publisher, the editors and the reviewers. Any product that may be evaluated in this article, or claim that may be made by its manufacturer, is not guaranteed or endorsed by the publisher.

## Abbreviations

ICU: Intensive Care Unit
SDU: Step-Down Unit
PPNR: Patient-Per-Nurse Ratio
HFNC: High Flow Nasal Cannulae
SARS-CoV-2: Severe Acute Respiratory Syndrome Coronavirus 2
SpO_2_: Oxygen Saturation
GW_C19_: General Ward Covid-19
LOS: Length-Of-Stay
SD: Standard Deviation
ICU_C19_: Intensive Care Unit Covid-19
ICU_no−C19_: Intensive Care Unit No Covid-19
HDU: High Dependency Unit
ED: Emergency Department.
